# An Atypical Local Vesicular Reaction to the Yellow Fever Vaccine

**DOI:** 10.3390/vaccines5030026

**Published:** 2017-09-19

**Authors:** Robert H. Wauters, Camellia L. Hernandez, Maureen M. Petersen

**Affiliations:** Walter Reed National Military Medical Center, Bethesda, MD 20889, USA; camellia.l.hernandez.mil@mail.mil (C.L.H.); maureen.m.petersen.mil@mail.mil (M.M.P.)

**Keywords:** yellow fever vaccine, adverse reaction, blisters, rash, hyperpigmentation

## Abstract

Yellow fever vaccine is a live attenuated viral inoculation indicated for patients traveling to endemic areas. The vaccine is generally well tolerated with minimal adverse effects. Typical side effects include malaise, pain at the injection site, and, albeit rarely, immediate hypersensitivity reactions. We present a case of a rare adverse reaction to yellow fever vaccine in which a patient developed vesicular lesions resulting in bullae and circumferential hyperpigmentation.

## 1. Case

A 31 year old female with no significant medical history presented to the travel clinic for guidance prior to travel to South Africa and Zimbabwe. Her travel plans also included a stop in Ethiopia requiring documentation of having received the yellow fever vaccine. She endorsed feeling well and at baseline health at the time of her evaluation. Her medical history was only notable for chronic rhinitis treated with loratadine and intranasal fluticasone. The patient was provided appropriate travel guidance and administered 0.5 mL of an intramuscular Sanofi Pasteur typhoid vaccine in the left deltoid and 0.5 mL of a subcutaneous Sanofi Pasteur yellow fever vaccine injected inferiorly and distal to the insertion of the deltoid muscle. The procedure was well tolerated with no immediate adverse events.

**Figure 1:**
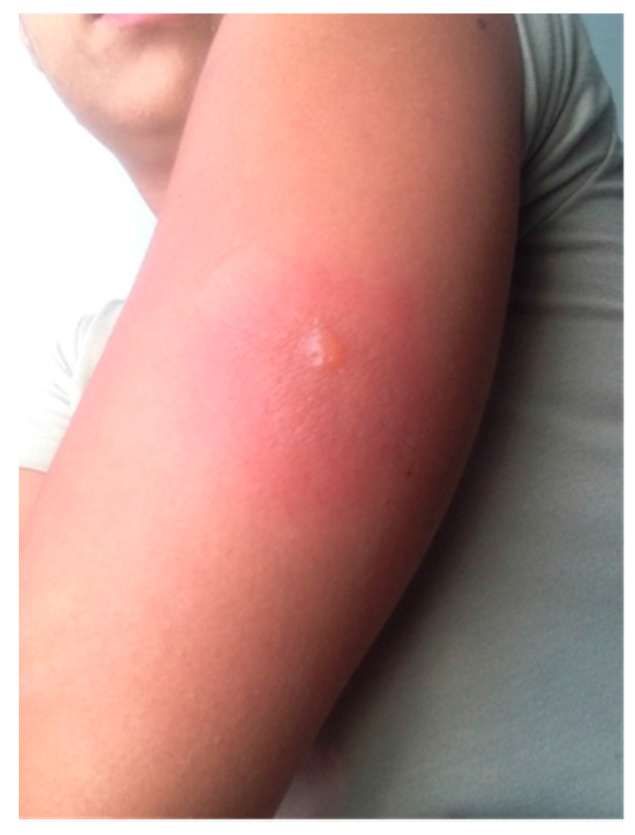
Day 2 following inoculation with initial formation of vesicles with surrounding erythema.

**Figure 2:**
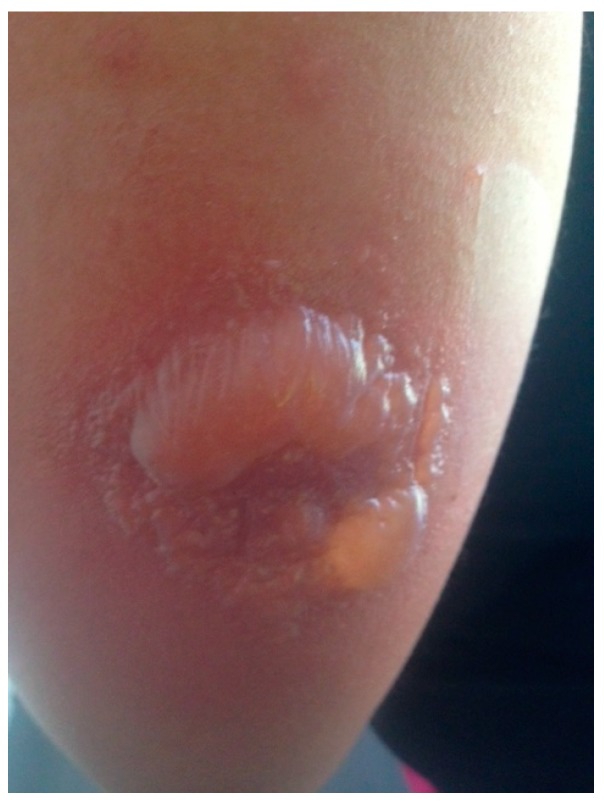
Day 5 after inoculation, the reaction site with multiple vesicles coalescing into a primary 4 cm bulla.

**Figure 3:**
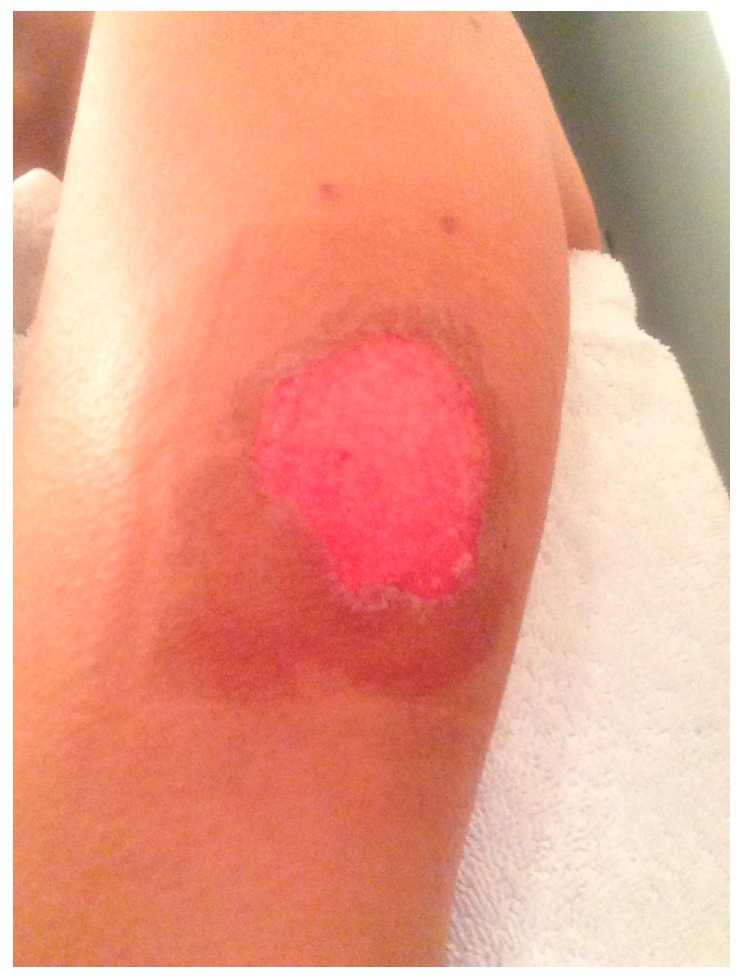
Day 7 post-inoculation with newly denuded skin with circumferential hyperpigmentation.

The day after inoculation, the patient noted a small blister overlying the yellow fever inoculation site (Figure 1). Over the ensuing days, multiple serosanguinous vesicles developed, with many coalescing into a primary 4 cm bulla. The area was pruritic and erythematous with underlying induration (Figure 2). The patient denied any systemic symptoms including fever. Five days post-vaccination she presented to a local emergency room where the bullous lesions were unroofed. She was discharged home with topical antibiotic ointment and advised to keep the area clean and covered. On the following day, the area surrounding the newly denuded skin was noted to be hyperpigmented (Figure 3).

The patient denied ever receiving a yellow fever vaccine and had no history of a reaction to any vaccine in the past, or history of contact dermatitis. She was closely monitored and had complete resolution of her symptoms within 3 weeks.

## 2. Discussion

Live attenuated yellow fever vaccines have been available since the 1930s and are generally considered safe and effective [1]. Known adverse systemic reactions to the yellow fever vaccine include headaches, myalgia, and low-grade fevers, which typically resolve in 5–10 days. Local reactions including edema, pain, and erythema are also common [2]. Rarely, more serious neurological adverse effects have been reported in the form of meningitis and meningio-encephalitis, with rates of occurrence as high as 1:10,000 [3]. Multi-organ failure, as defined by the Brighton Collaboration criteria, has also been identified as a rare adverse effect following yellow fever vaccination [4]. Anaphylaxis, though rare, has been seen in patients with a history of an allergy to eggs or other components of the vaccine [5]. Death has been reported at a rate of 0.1–2.6 per one million administered doses of yellow fever vaccine [6]. Our patient’s vesicular lesions with coalescence into bullae and hyperpigmentation is not a known or documented adverse reaction to the vaccine, and prompted further investigation using the Vaccine Adverse Event Reporting System (VAERS).

The VAERS database contains information on unverified reports of adverse events following immunization with US-licensed vaccines. VAERS was accessed using the Center for Disease Control Wide-Ranging Online Data for Epidemiologic Research (CDC WONDER) tool. The initial search of the entire database from its first year of tracking in 1990 through May 2017, yielded 30 case reports of a blistering reaction following administration of the yellow fever vaccine [7]. Meticulous review of each case identified 12 cases that noted the formation of vesicular lesions at the yellow fever inoculation site and 18 cases that reported vesicular lesions following the vaccine but occurring in areas other than the injection site.

The following discussion pertains to the 12 cases that reported vesicular formation near the inoculation site (Table 1). Case 1 described a small single vesicle occurring at the vaccine injection site. Cases 2–10 reported multiple vesicles occurring at the yellow fever inoculation site; however, no skin pigment changes developed or were reported. Cases 11 and 12 reported a reaction similar to our patient’s case. Case 11 described a patient who developed a large bulla with circumferential hyperpigmentation seven days after immunization. Case 12 described a patient with erythema, induration, and a coalescing bullous lesion at the inoculation site associated with surrounding hyperpigmentation. The time course of events in this case was not reported. The patient in Case 11 took no concomitant medications, and the patient in Case 12 took only fish oil and a daily multivitamin [7].

The possible etiologies of our patient’s reaction include a type IV hypersensitivity to the vaccine or its components. The yellow fever vaccine is known to contain live attenuated virus, gelatin, and sorbitol. A review of the literature identified multiple published reports of a gelatin hypersensitivity causing symptoms of anaphylaxis, but there has never been a published case report of a type IV hypersensitivity reaction to gelatin [8]. Sorbitol has also been implicated, albeit rarely, in type I hypersensitivity, but there are currently no known cases of a contact dermatitis to sorbitol [9,10,11,12]. Live viral replication and activity at the inoculation site was considered as a possible etiology of our patient’s symptoms; however, the bulla was unroofed and treated at an outside emergency room, and no specimen was taken for the purpose of reverse transcription polymerase chain reaction analysis. Our patient was concomitantly administered typhoid vaccination and was taking a second generation antihistamine, which we considered as potentially playing a role in her reaction. However, the two reported similar cases, Case 11 and 12, were not associated with antihistamine use or concomitant typhoid vaccination, and we think this is likely not an important contributing factor. It is unknown whether our patient became seropositive for yellow fever after her reaction. The patient is an active duty military service member and is no longer in the country. Additionally, it is not standard protocol to test for seroconversion in deploying forces.

## 3. Conclusions

Our patient’s case demonstrates a rare and significant adverse reaction to the yellow fever vaccine. Yellow fever is found in tropical and subtropical areas in South America and Africa, and travel to these areas warrants administration of the vaccine [13]. Early identification of vaccine adverse reactions, including rare and previously not described events, is important to health care providers in immunology, infectious disease, and primary care to ensure appropriate counseling of possible adverse events as well as prompt and appropriate treatment of these maladies.

## Figures and Tables

**Table 1:** 12 VAERS cases of local vesicular reaction to the yellow fever vaccine.

Case	VAERS ID	Description
1	041172-1	Single small blister
2	258521-1	Erythema, multiple vesicles, pruritus
3	261259-1	Vesicles, pruritus, erythema
4	306400-1	Erythema, multiple vesicles
5	391917-1	Erythema, multiple vesicles
6	419936-1	Erythema, multiple vesicles
7	421959-1	Vesicles, pruritus
8	453003-1	Erythema, multiple vesicles, pruritus
9	559035-1	Vesicles, induration, erythema
10	337338-1	Erythema, large vesicle
11	390002-1	Bulla, circumferential hyperpigmentation 7 days after vaccination
12	471071-1	Coalescing vesicles, hyperpigmentation
